# FLARE: a fast and flexible workflow for identifying RNA editing foci

**DOI:** 10.1186/s12859-023-05452-4

**Published:** 2023-10-02

**Authors:** Eric Kofman, Brian Yee, Hugo C. Medina-Munoz, Gene W. Yeo

**Affiliations:** 1 Department of Cellular and Molecular Medicine, University of California San Diego, La Jolla, CA USA; 2 Sanford Stem Cell Institute Innovation Center and Stem Cell Program, University of California San Diego, La Jolla, CA USA; 3 Institute for Genomic Medicine, University of California San Diego, La Jolla, CA USA

**Keywords:** RNA editing, Clustering, Statistical, Modeling

## Abstract

**Background:**

Fusion of RNA-binding proteins (RBPs) to RNA base-editing enzymes (such as APOBEC1 or ADAR) has emerged as a powerful tool for the discovery of RBP binding sites. However, current methods that analyze sequencing data from RNA-base editing experiments are vulnerable to false positives due to off-target editing, genetic variation and sequencing errors.

**Results:**

We present FLagging Areas of RNA-editing Enrichment (FLARE), a Snakemake-based pipeline that builds on the outputs of the SAILOR edit site discovery tool to identify regions statistically enriched for RNA editing. FLARE can be configured to analyze any type of RNA editing, including C to U and A to I. We applied FLARE to C-to-U editing data from a RBFOX2-APOBEC1 STAMP experiment, to show that our approach attains high specificity for detecting RBFOX2 binding sites. We also applied FLARE to detect regions of exogenously introduced as well as endogenous A-to-I editing.

**Conclusions:**

FLARE is a fast and flexible workflow that identifies significantly edited regions from RNA-seq data. The FLARE codebase is available at https://github.com/YeoLab/FLARE.

**Supplementary Information:**

The online version contains supplementary material available at 10.1186/s12859-023-05452-4.

## Background

Transcriptomics assays that leverage RNA base editing, such as DART-seq [14], TRIBE [13] and STAMP [3] have recently gained visibility as alternative and complementary technologies to immunoprecipitation-based methods in the mapping of the binding sites of RNA-binding proteins (RBPs). In general, such approaches involve the expression of a chimeric protein containing an RNA-editing enzyme and the RBP under investigation such that upon sequencing, the resulting bioinformatically identified edits indicate loci where the RBP interacts with transcripts. Compared to traditional cross-linking and immunoprecipitation (CLIP)-based technologies, editing-based technologies offer advantages such as lower input material and faster and technically simpler assays, enabling higher throughput RBP binding site analyses with isoform sensitivity and utility in single cell assays [3]. Although these novel technologies continue to be optimized for uses across a wide variety of RBPs, cell types and conditions, it is clear that such a high-throughput protocol needs to be paired with a high-throughput, scalable and automated pipeline for determining sites with significant editing, which we call “clusters.” Calling clusters from edit data requires separating true edits from noise, with such noise deriving either from sequencing errors or off-target editing. While there are many tools available for detecting RNA-editing sites (i.e. single bases experiences editing), including SAILOR [4], SPRINT [24], and REDItools [12], there currently exist no computational pipelines for detecting clusters of enriched editing across groups of sites, aside from RNAEditor [8], which only is applicable for detection of A-to-I edits.

To address this need to identify regions exhibiting significant enrichment for various types of RNA edits, we present the FLARE (FLagging Areas of RNA-editing Enrichment) analysis pipeline. FLARE allows for analyses of all types of RNA base changes, including A-to-I, C-to-U, and U-to-C [7], with only minor changes in configuration required to enable analysis of additional types of synthetic RNA edits. Using SAILOR outputs as a starting point—although in principle outputs from other similar tools like JACUSA2 [18] could be adapted for use as FLARE inputs—FLARE accounts for background editing rates to filter false positives from truly edited regions, and scores identified clusters for use in downstream applications.

We demonstrate FLARE’s application to analyzing C-to-U RNA-editing data in the context of STAMP using the RNA-binding protein RBFOX2, in A-to-I editing in the context of TRIBE, and in the context of endogenous ADAR-deposited A-to-I edits. We also include a Snakemake [15] implementation of the SAILOR software [4] updated to enable detection of any types of edits.

## Implementation

### Region windowing, edit tabulation and Poisson filtering

**Fig. 1:**
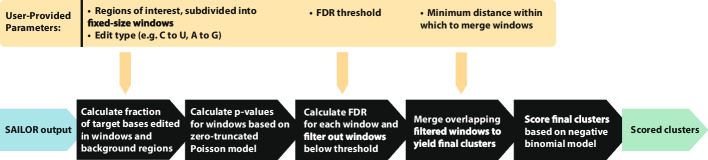
FLARE pipeline schematic. SAILOR edit sites serve as anchors informing which regions to search for edit enrichment. After filtering regions based on edit fraction using a Poisson model, remaining regions are merged to yield final clusters, which are then scored for downstream use

FLARE takes as inputs the edited positions, which are the sites that contain at least one edit conversion at that position within any read as determined by the SAILOR algorithm [4]. The core assumption underlying FLARE’s methodology is that a large fraction of these candidates, after the removal of common SNPs as part of the SAILOR workflow, might represent non-specific binding or sequencing errors. These should be filtered out in the process of identifying regions where an RNA-editing enzyme, whether fused to an RBP as in STAMP or not, is more consistently editing. FLARE subdivides any gene sequences containing edits into fixed-size tiled windows in which the fraction of editable bases that are edited is calculated (a script for generating appropriate windows from a Gencode [5] GTF (Gene transfer format) file is included in the FLARE codebase). Applying a simplifying assumption that RNA-editing enzymes are uniformly likely to non-specifically stochastically interact with all expressed transcripts, we frame the deposition of spurious “noisy” edits found within each window as a Poisson process, with the Poisson rate parameter representing how many such “background” edits per editable site might be expected on average. Editable sites are defined as, for example, all Cs in a window in the case of C-to-U editing. Any windows exhibiting a statistically significant increase in their edit counts relative to the expected, Poisson-modeled distribution of “noisy” edits are considered enriched for editing, and kept for further processing (Fig. 1).

Given that FLARE only considers genomic regions known to have at least one edited site, we employ a zero-truncated Poisson model, which enforces a 0% probability of a given window having zero edits. Simulating edit count data for one replicate using this model, based on the number of editable bases in a window, lines up well with empirical data (Additional file 1: Figure S1A). Adjusted residuals between modeled and empirical edit counts are only slightly positively correlated with total target editable substrates, indicating that the Poisson process is a reasonable model (Additional file 1: Figure S1B).

Clearly, the choice of the Poisson parameter is important—if it is too high this will lead to an increase in false negatives because certain regions that generally see lower editing will not achieve statistical significance, while if it is too low this will instead lead to an increase in false positives. FLARE employs a mix of different Poisson parameters for different subsets of windows to best account for region and coverage-related variation in background editing rates.

The first distinction is made between editing in intronic and exonic regions. RNA background editing rates would be expected to differ across intronic and exonic regions depending on where in the cell and at what point during pre-mRNA processing editing occurs. For example, a cytoplasmic RBP might be expected to interact less with intronic regions than a nuclear RBP, and any RNA base-editing enzyme fused to such an RBP would naturally deposit edits differentially on such regions. FLARE explicitly incorporates this possible difference in exonic and intronic editing noise, using the average editing rate across only all exons as the Poisson parameter when processing exons, and likewise for introns.

In addition, low read depth in a given window can mean that edit fractions are artificially inflated by noise, and must be taken into account when calculating a Poisson parameter. For instance, in a window where there are only 5 possible substrate bases due to low coverage, a single edited base will lead to a window edit fraction of 0.20, whereas a single edit in a window with 500 substrate bases will have an edit fraction of 0.002. Any window containing a single edit in a single read bearing multiple possible editable sites arguably represents noise, and it is evident that evaluating all windows using a Poisson background model derived from the mean edit fraction of highly covered windows will lead to an excess of such lowly covered windows being called significant.

Assuming that edits accumulate at regions where the RBFOX2 fusion protein is present, one would generally expect windows with higher edit fractions to be more reflective of authentic RBFOX2 binding sites. That is, they should be more likely to overlap with RBFOX2 eCLIP (enhanced cross-linking immunoprecipitation) peaks, and to contain the canonical GCAUG RBFOX2 binding motif, irrespective of window read depth. However, the correlation of edit fraction with these two “ground truth” metrics only approaches steady-state value for windows with at least 50 reads in our example (Additional file 2: Figure S2A). Based on this observation, the Poisson parameter for windows with lower read depths is calculated by taking the mean edit fraction of only windows with lower read depths. More specifically, a separate Poisson parameter is calculated for windows with less than 10, 20, 30, 40 and 50 reads, respectively. This ensures that the signal strength at lowly covered regions must be stronger than that at highly covered regions for a window to be considered significant.

### False discovery rate calculation and window merging

After windows are assigned p-values depending on their particular Poisson model, multiple hypothesis testing is accounted for by applying a Benjamini–Hochberg false discovery rate procedure to generate adjusted p-values for all windows. Contiguous windows with an adjusted p-value exceeding a user-defined filtering threshold (default of 0.1) are then merged to form clusters. Window-merging distance, which defaults to 15 base pairs, can be increased by users desiring coarser clusters (Fig. 1).

### Cluster scoring

Once final clusters have been obtained by merging filtered windows, the FLARE pipeline assigns them a score based on the cumulative distribution function (CDF) value of a given cluster edit fraction within a negative binomial distribution, using mean editing fraction among clusters as the *p* parameter.

## Results

### FLARE interface and usage

The FLARE pipeline is command-line based, and uses the Python-based pipelining framework Snakemake [15]. All processing steps described above run automatically to completion when all required inputs—a GTF file of window regions, a reference fasta file, and bam file, bigwigs and SAILOR output for a given sample—are present. Required libraries are loaded automatically using Snakemake’s Singularity [10] integration, ensuring portability across Linux releases at least as new as CentOS 7. The current FLARE release also includes an updated Snakemake version of the SAILOR [4] pipeline—future releases will merge the SAILOR and FLARE pipelines to enable automatic runs all the way from fastq files to clusters.

### FLARE filters out editing noise

We initially tested FLARE on RBFOX2-APOBEC1 STAMP data (C-to-U edits) in HEK293T cells, which were transiently transfected with RBFOX2-APOBEC1 fusion construct (doxycycline-induced expression for 72 h) before being prepared for RNA sequencing at a depth of 30 million reads. Based on our experience, we recommend that any library being used for RNA editing analyses with SAILOR and FLARE be sequenced at least this deeply.

**Fig. 2:**
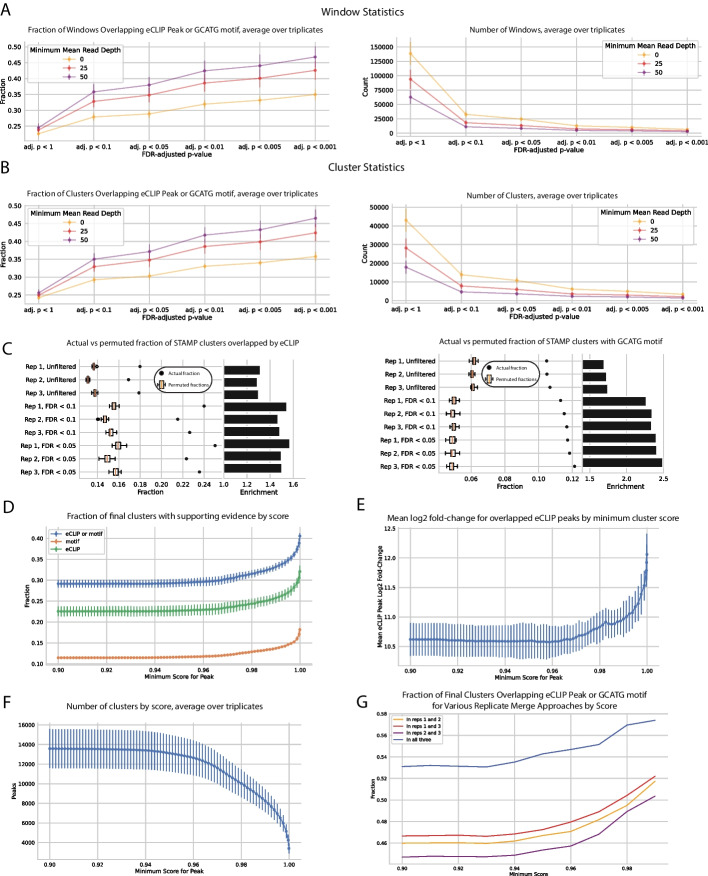
Poisson model scoring filters out regions less likely to reflect true RBFOX2 binding events.** a** Windows with lower adjusted p-values are more likely to overlap with the canonical RBFOX2 motif, GCAUG, and with eCLIP peaks.** b** Clusters resulting from merging filtered windows reflect a similar trend, with clusters formed from more stringently filtered windows exhibiting a larger overlap with both motif and eCLIP peak presence.** c** Clusters formed from more stringently filtered windows are more highly enriched for motif and eCLIP presence compared to a null distribution of clusters of comparable sizes randomly shuffled within respective introns and exons.** d** RBFOX2 clusters with higher scores exhibit higher overlap with the canonical GCAUG motif or RBFOX2 eCLIP peaks.** e** Among clusters that overlap eCLIP peaks, higher scoring clusters tend to overlap stronger eCLIP peaks.** f** Representation of the number of clusters by score.** g** Cluster quality can be boosted immensely by retaining clusters found across multiple biological replicates

In this RBFOX2 example, window and cluster specificity can be evaluated using eCLIP peak and canonical RBFOX2 motif (GCAUG) overlap, under the assumption that true binding sites should have a higher overlap with such features. The publicly available eCLIP dataset we used for this calculation [3] represents binding sites of the chimeric RBFOX2-APOBEC1 protein, filtered for peaks with *log2(fold-change)* > *1* and *p-value* < *0.05*. By our overlap metric, it is clear that applying more stringent adjusted p-value filters results in better window filtering and higher quality clusters, regardless of window read depth. More specifically, 28%, 32% and 35% of windows with adjusted p-values of below 0.1, 0.01, and 0.001 overlapped with eCLIP peaks or the canonical motif, respectively, with these proportions increasing to 36%, 42% and 47% for windows with a read depth exceeding 50 (Fig. 2A). After merging, these windows yielded clusters exhibiting similar overlap fractions of 29%, 33% and 36%, respectively; clusters derived from windows with at least 50 reads exhibited overlap fractions of 35%, 42%, and 47% (Fig. 2B).

This increase in cluster quality corresponding to increasing p-value filtering stringency indicates that our model is successfully preferentially filtering out false positive sites. Importantly, using FLARE’s window-based approach to cluster detection attains higher sensitivity and specificity (more clusters at a lower false positive rate) than merely filtering individual SAILOR-called sites based on SAILOR confidence scores (Additional file 2: Figures S2B, S2C).

Of course, the more genomic area any putative clusters cover, the more they will trivially overlap eCLIP peaks and canonical motifs, so it is important to ensure that the overlap is statistically significant. To this end, we randomly relocated clusters within their respective exons to create 30 artificial cluster sets derived from each replicate. We observed that none of these permutations coincide with eCLIP peaks and with the canonical GCAUG RBFOX2 motif as frequently as the true experimental clusters (Fig. 2C). In addition, increasing adjusted p-value filtering stringency also increases the overlap enrichment of our clusters compared to the artificial shuffles, defined as the true overlap value divided by the mean overlap value of the random permutations. This empirical assessment suggests that FLARE clusters are enriched for eCLIP overlap and GCAUG content with a high degree of significance, and that increasing FLARE filtering stringency enhances this enrichment.

### FLARE scores stratify edited regions by confidence

Cluster specificity can be improved by retaining clusters with higher scores, which are more likely to overlap eCLIP peaks and GCAUG motifs (Fig. 2D). In addition, among the clusters that overlap eCLIP peaks, higher scoring clusters tend to overlap stronger eCLIP peaks (based on eCLIP log2 fold-change), implying that cluster score could possibly be an indicator of binding strength (Fig. 2E). Naturally, filtering more stringently comes at the cost of sensitivity, with less clusters recovered overall at higher minimum score thresholds (Fig. 2F). Choosing an appropriate threshold depends on how many clusters are identified by FLARE and how much stringency is desired, but based on this example we recommend filtering for clusters with a score of at least 0.95 initially.

### Use of replicates and further exploration of FLARE cluster properties

The filtering and scoring steps in the FLARE pipeline successfully separate signal from noise, but confirmation of cluster presence across multiple replicates is recommended to further improve precision and confidence in detected binding-sites. We see a vast improvement in specificity attained in this example when retaining only clusters found across all 3 biological replicates, with up to 58% of the highest-scoring clusters overlapping with eCLIP or the canonical GCAUG motif (Fig. 2G).

There are various plausible reasons why even after filtering for high FLARE scores there is still a subset of STAMP editing clusters that overlap neither eCLIP peaks nor the canonical GCAUG motif. First of all, the APOBEC1 catalytic site does not edit at the actual binding site, which is naturally occupied by RBFOX2. It follows that many clusters should be expected to reside at some distance from true binding sites, as Rahman et al. described in the context of their similar HyperTRIBE A-to-I editing-based method [21]. Indeed, many eCLIP peaks and GCAUG motifs can be found in regions within 200 bases from cluster boundaries, which might explain the presence of some of STAMP-specific clusters (Additional file 2: Figure S2D).

Along with this, introduction of the APOBEC1-RBFOX2 construct effectively results in elevated RBFOX2 concentrations, so that the fusion construct may be more likely to bind to lower binding-affinity, secondary versions of the GCAUG motif, as Begg et al. [2] described in their 2021 publication. To see if these intermediate affinity motifs could partially explain some FLARE clusters’ provenance, we calculated what fraction of clusters contain at least one of the 7 top motifs found by Begg et al. [2] to most strongly bind RBFOX2: GCACG, GCUUG, GAAUG, GUUUG, GUAUG, GUGUG and GCCUG. Compared to a set of control motifs (all possible pentamers, excluding ones containing 3-mer substrings of these 7 RBFOX2 motifs, or the highly conserved start and end pair of Gs), eCLIP-overlapping clusters are slightly more likely than non eCLIP-overlapping clusters to contain secondary motifs (Mann–Whitney U test p-value = 0.038; Additional file 2: Figure S2E), indicating that the presence of an RBFOX2 secondary motif can increase our confidence that the cluster reflects a real binding event. Even so, the fraction of eCLIP-overlapping clusters containing such motifs (Additional file 2: Figure S2F) is of a similar magnitude to that in non-eCLIP-overlapping clusters (Additional file 2: Figure S2G), relative to the control background, thus we can infer that many of these FLARE-exclusive clusters may indeed reflect real binding events at intermediate affinity motifs. Under this assumption, then over 80% of FLARE clusters can be considered to have eCLIP or motif-based evidence.

For reference, a majority of FLARE clusters in this example are less than 150 bases long, with a long tail of longer clusters (Additional file 2: Figure S2H), and only between 25 and 40% of individual edit sites are found in final clusters, depending on the score threshold used (Additional file 2: Figure S2I). Given the boost in specificity attained at higher score thresholds, this relatively low edit-in-cluster fraction simply reflects the high levels of off-target noise expected in a dataset of this nature and reinforces the need for statistical filtering.

Window size does affect final clusters and can be tuned empirically to achieve optimal results. In these analyses we use a window size of 30 base pairs (set as the default in the window-generating script), as it appears to strike a balance between precision and statistical significance among the 10, 20, 30, 40, and 50 base pair size options we tested (Additional file 4: Figure S4A, B, C, D). In general, an optimal clustering solution will maximize the average number of editing sites per cluster, which should contain more than one editing site, while minimizing the total genomic area covered by all clusters, which ensures that cluster coordinates are precise enough to be useful in ascertaining exact binding sites. When assessing different window sizes, we suggest using a metric we term the “efficiency score”, (Additional file 4: Figure S4E), which incorporates these two competing demands into one optimization problem; in our example, we do indeed observe a maximization of the efficiency score at a window size of 30 base pairs. As 30 base pairs also appears to work well in the analyses described in the next sections, we recommend it as a reasonable window size for most analyses.

### Application to TRIBE and HyperTRIBE A-to-I editing data

**Fig. 3:**
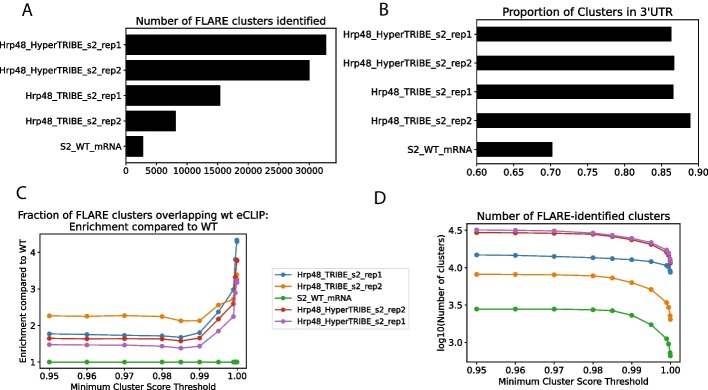
FLARE can be used to analyze A-to-I editing in TRIBE and HyperTRIBE data. **A** An order of magnitude more clusters are found in Hrp48-HyperTRIBE datasets than in Hrp48-TRIBE datasets, which in turn has an order of magnitude more clusters than the WT control. **B** A greater proportion of clusters are found in 3’UTRs in the TRIBE and HyperTRIBE datasets than in the WT datset. **C** The relative differences in cluster count are preserved regardless of what FLARE score threshold is used. **D** Higher FLARE scores correlate positively with fraction of clusters overlapping CLIP peaks, normalized to fraction for WT

To demonstrate FLARE’s compatibility with methods that use A-to-I editors, we ran SAILOR and FLARE on ADAR-induced editing data produced by Hrp48-TRIBE and HyperTRIBE in S2 cell lines [13, 21]. As expected, based on the known improvements in editing exhibited by HyperTRIBE, FLARE identifies an order of magnitude more clusters in the Hrp48-HyperTRIBE dataset than in the Hrp48-TRIBE data, which in turn has an order of magnitude more clusters than a wild-type control (Fig. 3A). As described in McMahon et al. [13], a greater proportion of these clusters are found in the 3’UTRs than in the WT control (Fig. 3B). In addition, the differences in editing cluster counts are preserved across all FLARE cluster score thresholds (Fig. 3C), with FLARE scores for these clusters correlating well with enrichment of clusters for overlap with Hrp48 CLIP (Fig. 3D), both when comparing to wild-type Hrp48 CLIP data and Hrp48-TRIBE CLIP data (Additional file 3: Figure S3A). We have demonstrated how FLARE can be used to assess both C-to-U and A-to-I based editing data in STAMP and TRIBE-like systems, but in principle SAILOR and FLARE are also amenable to analyses of any other type of base conversion.

### Application to native ADAR A-to-I editing cluster detection

To assess to what extent FLARE can also be applied to detection of naturally occurring RNA editing, we ran FLARE on RNA-seq data from U87 glioblastoma cell lines from a recent publication exploring ADAR3’s impact on RNA A-to-I editing in glioblastoma [20]. Adenosine-to-Inosine edits, which are catalyzed by members of the ADAR family on double-stranded RNA (dsRNA) substrates, are in coding regions usually found at isolated single sites. However, in non-coding areas of the genome including introns, 3’ UTRs and intergenic regions, they instead tend to be found in dense clusters amenable to detection using the FLARE methodology—indeed, this applies for more than 99% of the millions of A-to-I edits in the genome [1]. More specifically, edits caused by ADAR1 are highly likely to be found in *Alu* repeat elements, which naturally form dsRNA due to their palindromic nature [1, 9].

Decreases in *Alu* editing have been observed across a variety of cancer tissues, both in terms of numbers of edited sites and fraction of reads edited at such sites [16]. In the brain, this inhibition of editing is in some cases prompted by the binding of ADAR3, which, unlike its globally expressed family members ADAR1 and ADAR2, is specifically expressed in brain tissue and does not exhibit A-to-I editing activity [19].

In a recent publication comparing A-to-I editing in U87 glioblastoma cells (which endogenously express low levels of ADAR3) to editing in U87 cells with higher levels of ADAR3 exogenously introduced, Kurup et al. determined that transcripts exhibiting reduced A-to-I editing in glioblastoma are more likely to be bound to ADAR3. Within such transcripts, Kurup et al. identified several dozen genomic sites consistently exhibiting reduced editing across three biological replicates, but note that apart from these specific instances, “there was substantial variability in the specific sites with altered editing.” Indeed, among the replicates, about ten times more differentially edited sites were only specific to one replicate than were shared. Examining editing changes at wider regions representative of clustered editing, using the approach offered by FLARE, enables the aggregation of RNA editing signals to elucidate interesting changes that might be missed when analyzing changes at a site-specific level.

### Comparing changes in A-I editing rates in FLARE-identified clusters across conditions

**Fig. 4:**
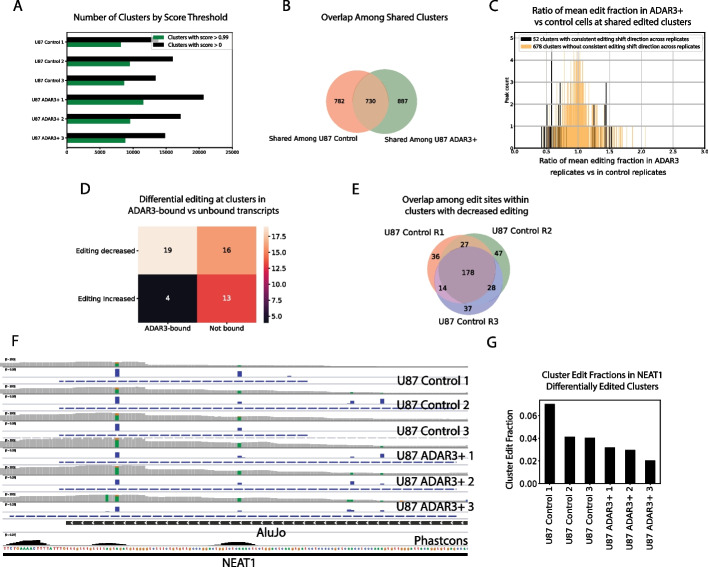
FLARE identifies regions with ADAR3-linked differential A-to-I editing.** a** Number of A-to-I editing clusters called across samples, without and with minimum score filtering.** b** Shared replicated clusters distribution.** c** Histogram depicting ratio of mean edit fraction in ADAR3+ vs control U87 at shared editing clusters identified by FLARE.** d** Shared clusters bound by ADAR3 are highly enriched for a decrease in edit fraction in the ADAR3+ condition compared to the control condition.** e** Even among clusters shared by replicates, many editing sites are unique to each replicate.** f** IGV snapshot of a Alu-overlapping region towards the 3’ end of non-coding RNA NEAT1 which experiences reduced editing in an ADAR3+ context. Coverage, edit fraction, and cluster regions plots are shown for each sample, with edits highlighted on bar plots (“A”s are green and “G”s are brown). A phastcons (20 species) conservation track is also shown at bottom. **g** Edit fractions across samples, within the differentially edited NEAT1 cluster

Running SAILOR followed by FLARE on the three wild-type (WT) replicates and ADAR3 + replicates yielded several thousand clusters across all six samples (Fig. 4A). To ensure subsequent analyses were carried out on highly confident clusters, the clusters with a score below 0.99 were filtered out, which reduced the total cluster count by about half across samples (Fig. 4A). Among shared clusters within the WT set, correlation coefficients of cluster edit fraction between any two of the three replicates ranged from 0.93 to 0.94, and among shared clusters within the ADAR3 + set, correlation coefficients ranged from 0.86 to 0.9. This consistency supports the assertion that the clusters represent real A-to-I editing loci. There were 730 clusters exhibiting A-to-I editing that were shared across all replicates in both conditions, among which slightly less than 10% exhibited a directionally consistent shift in edit fraction between conditions (Fig. 4B, C). Echoing Kurup et al.’s findings in their original single site analysis, overlapping clusters with decreased editing were more likely to be found in transcripts experimentally found to be bound by ADAR3 than overlapping clusters without decreases in editing (p-value = 0.0346, one-tailed Fisher’s Exact test; Fig. 4D), supporting the idea that ADAR3 presence on transcripts can inhibit the editing activity of ADAR1. It is worth noting that many individual editing sites within clusters exhibiting decreased editing are not shared across replicates, which would preclude the identification of a subset of these differentially edited loci using a site-specific approach (Fig. 4E).

While a cluster-based approach still recapitulates the main finding in Kurup et al. that the innate immunity-related protein MAVS exhibits reduced editing, there were also many other interesting genes that emerged from this analysis that were not found across all replicates in Kurup et al.’s site-specific approach. Specifically, a region coinciding with an *AluJo-* element towards the 3’ end of the non-coding RNA NEAT1, whose expression has been linked to tumor malignancy in glioma [6, 11, 23], was found to consistently experience less editing in the ADAR3 + condition than in the control condition (Fig. 4F, G). Intriguingly, a recent study found that the NEAT1 transcript is stabilized via changes in binding behavior enabled by ADAR1-induced RNA editing, where the A-to-I edits in question are found within a dsRNA structure involving the exact same *Alu* element identified using FLARE [22]. Given that NEAT1 editing is inhibited in the ADAR3 + condition, it might be expected that reduced ADAR3 levels might stabilize NEAT1 and contribute to tumor growth. This effect is seen in glioma, with ADAR3 expression dropping as tumor stage progresses, a pattern found both in the Chinese Glioma Genome Atlas (CGGA) and The Cancer Genome Atlas (TCGA; [25]). Although NEAT1 was not particularly highly enriched for binding to ADAR3 in Kurup et al.’s RIP-seq assay, it was nevertheless enriched in IP fractions compared to input fractions in the ADAR3 + condition, leaving open the possibility of ADAR3-NEAT1 interactions. While follow-up experiments are necessary for confirmation, it is plausible that editing changes in the differentially edited region identified in NEAT1 using a cluster-based approach may be linked to tumor progression.

In general, the results of running FLARE to identify A-to-I editing clusters in these U87 samples indicates that the pipeline can indeed be successfully applied to detect endogenous RNA editing, and that employing a cluster-based method highlights different areas than a site-specific method when looking at differential editing. When analyzing clustered editing sites in non-coding regions of the genome, such as in *Alu* regions, a cluster-based approach may serve as an informative complement to a site-based approach.

### Comparison to similar tools

The most similar available pipeline for highlighting edit clusters is RNAEditor [8], which is designed to exclusively detect A-to-I edits, and finds RNA editing “islands” using heuristic cutoffs of edited site merge distance and cluster minimum edit site counts. We benchmarked FLARE’s performance against RNAEditor’s to make sure it achieves comparable results. We ran FLARE on the same datasets from the original RNAEditor publication, namely whole transcriptomes of B cells both treated with non-targeting control (“NT”) and ADAR1 siRNAs.

**Fig. 5:**
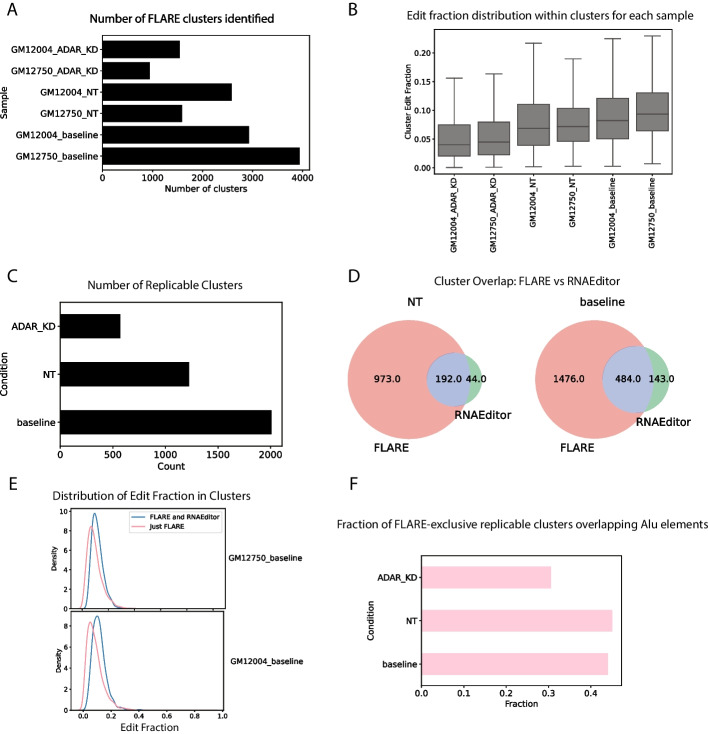
Comparison of FLARE to RNAEditor. **A** ADAR1-knockdown leads to a decrease in cluster counts **B** Clusters in the ADAR1-knockdown condition have lower edit fractions. **C** Replicable clusters also conform to the expected trend. **D** FLARE recapitulates a majority of RNAEditor “editing islands.” **E** The additional clusters found by FLARE but not RNAEditor tend to be of lower edit fraction, reflecting FLARE’s increased sensitivity. **F** FLARE-exclusive clusters exhibit the expected decrease in overlap fraction with *Alu* elements

As a minimum validation, after filtering for clusters with the maximum scores, FLARE-derived results recapitulate John et al.’s expected observation that knocking down ADAR1 leads to fewer editing clusters (Fig. 5A) [8]. In addition, the clusters detected by FLARE in the ADAR1-knockdown condition generally boast lower edit fractions than those in the baseline or non-targeting siRNA conditions, again as expected (Fig. 5B), and replicable clusters for each condition also exhibit the expected count decrease (Fig. 5C).

Next we compared the overlap in A-to-I editing clusters found by each approach. In the baseline (untreated) condition, which had the most RNAEditor editing clusters, replicable FLARE clusters coincided with 484 of 627 (77.2%) replicable RNAEditor clusters (Fig. 5D). However, FLARE appears to be more sensitive, finding a total of 1960 or approximately three times more replicable clusters than RNAEditor. Indeed, the replicable clusters exclusively found by FLARE tended to be of lower editing fractions (Fig. 5E); RNAEditor’s heuristic approach might have missed them. To address the possibility that the clusters found exclusively by FLARE were not false positives, we demonstrated that the FLARE-specific clusters exhibited a marked decrease in overlap with *Alu* elements in the ADAR1 knockdown condition compared to the non-targeting and baseline conditions (Fig. 5F). Given that ADAR1 is known to highly edit Alu elements [17], this lends weight to the argument that they are not false positives, and most likely do reflect true ADAR1-edited sites. Overall, this comparison again demonstrates that FLARE is well-suited to analysis of endogenous A-to-I editing, and that it achieves similar results to approaches currently in use.

## Conclusion

Whether applied in the context of exogenous RNA-editing using technologies such as STAMP or TRIBE, or endogenous ADAR-based A-to-I editing, the FLARE pipeline is an effective tool to efficiently locate and prioritize loci significantly enriched for editing. We demonstrate that FLARE’s Poisson-based filtering approach preferentially filters out false positive editing sites to yield high confidence clusters, and that the scores FLARE subsequently assigns to these clusters enable further stratification and increased stringency.

We emphasize that FLARE is capable of detecting clusters of any type of transition or transversion—to our knowledge, there are no existing RNA editing site detection and clustering pipelines currently available that can process all edit types.

FLARE leverages the Snakemake python framework [15] and Singularity [10] to ensure that researchers can easily run each step of FLARE on their data without worrying about package requirements or obscure mid-run failures. FLARE is a parallelizable, fast and easily implementable tool for detecting editing-enriched foci, and we anticipate that it will prove useful to those in the research community looking to gain more insights from their RNA editing data.

### Supplementary Information

**Additional file 1: Figure S1.** Validation of the zero-truncated Poisson model. **a** Simulated edit count distribution matches actual edit count distribution well. **b** Residuals at lower coverage windows exhibit little correlation to coverage. It is worth noting that although the model loses fidelity in regions at extremely highly covered windows (**c**), the resulting overestimation of expected edit counts in such areas will tend to lead to under-calling of regions rather than false positives, erring on the conservative side to increase precision at the expense of recall

**Additional file 2: Figure S2.** FLARE cluster properties and comparison to site-based approach. **a** The correlation of window edit fraction with window precision, which is expected to be both positive and uncorrelated to window read depth, only stabilizes above a read depth of approximately 50 reads. **b** Generating 50 base pair windows surrounding individual SAILOR sites, filtered by individual SAILOR site score exclusively, results in lower specificity than the approach employed by FLARE. **c** A site-based approach also yields lower sensitivity than the cluster-based approach. **d** Expanding RBOX2 FLARE clusters leads to higher rates of eCLIP or canonical RBFOX2 motif overlap. **e** FLARE clusters overlapping eCLIP are slightly likelier than ones not overlapping eCLIP to contain secondary RBFOX2 motifs. **f** A higher fraction of eCLIP overlapping clusters contain secondary motifs than control motifs. **g** A higher fraction of non-eCLIP overlapping clusters contain secondary motifs than control motifs. **h** Between approximately 25% and 40% of edit sites can be found within peaks depending on score threshold. **i** The cluster length distribution with 30 bp windows has a long tail but is centered around approximately 100 bp

**Additional file 3: Figure S3.** TRIBE and HyperTRIBE datasets are amenable to FLARE analysis. **a** There is an enrichment for overlap with Hrp48 CLIP peaks of Hrp48-TRIBE and Hrp48-HyperTRIBE, when normalized to WT overlap

**Additional file 4: Figure S4.** Window size affects sensitivity and statistical significance—graphs produced using a single replicate. **a** Larger windows yield more precise but slightly less statistically significant RBFOX2 clusters based on eCLIP overlap. **b** Larger windows yield more precise but far less statistically significant RBFOX2 clusters based on eCLIP overlap. **c** Marginal gains in precision from larger window sizes decrease for each size increase. **d** As window size increases, neighboring small clusters merge to form larger clusters, but this merging and the resultant decrease in total clusters is less dramatic at higher values. **e** The “efficiency score” metric can be serve as a guide to tune window sizes for optimal clustering

## Data Availability

The datasets used and/or analyzed during the current study are available through GEO at accession numbers GSE215252 and GSE166402.

## Abbreviation

RBP: RNA-binding protein
