# Supplementary figures and images for: FLARE: a fast and flexible workflow for identifying RNA editing foci
Source: BMC Bioinformatics. 2023 Oct 2;24:370. doi: 10.1186/s12859-023-05452-4 (PMC10544219; doi:10.1186/s12859-023-05452-4)

A

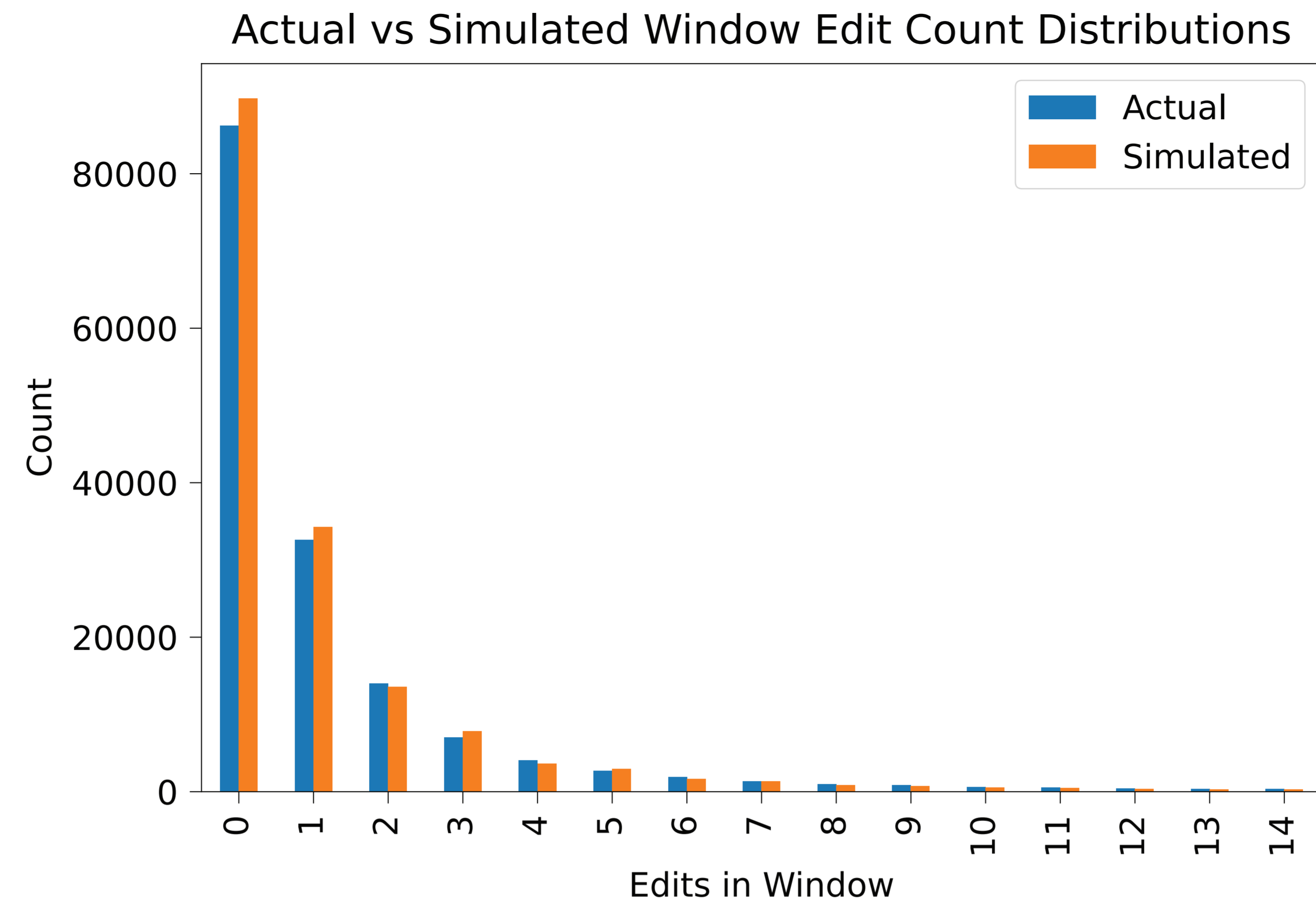

B

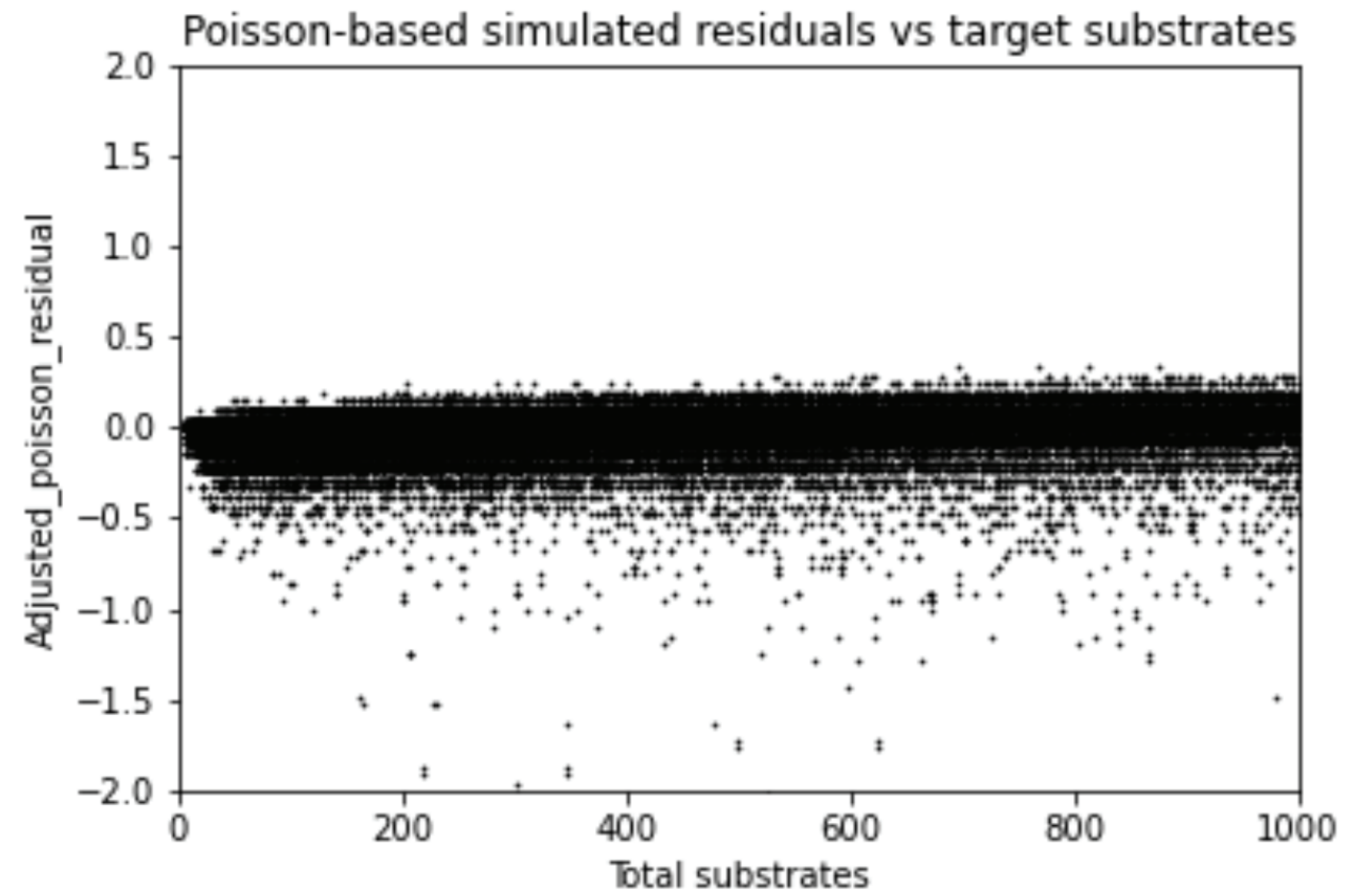

C

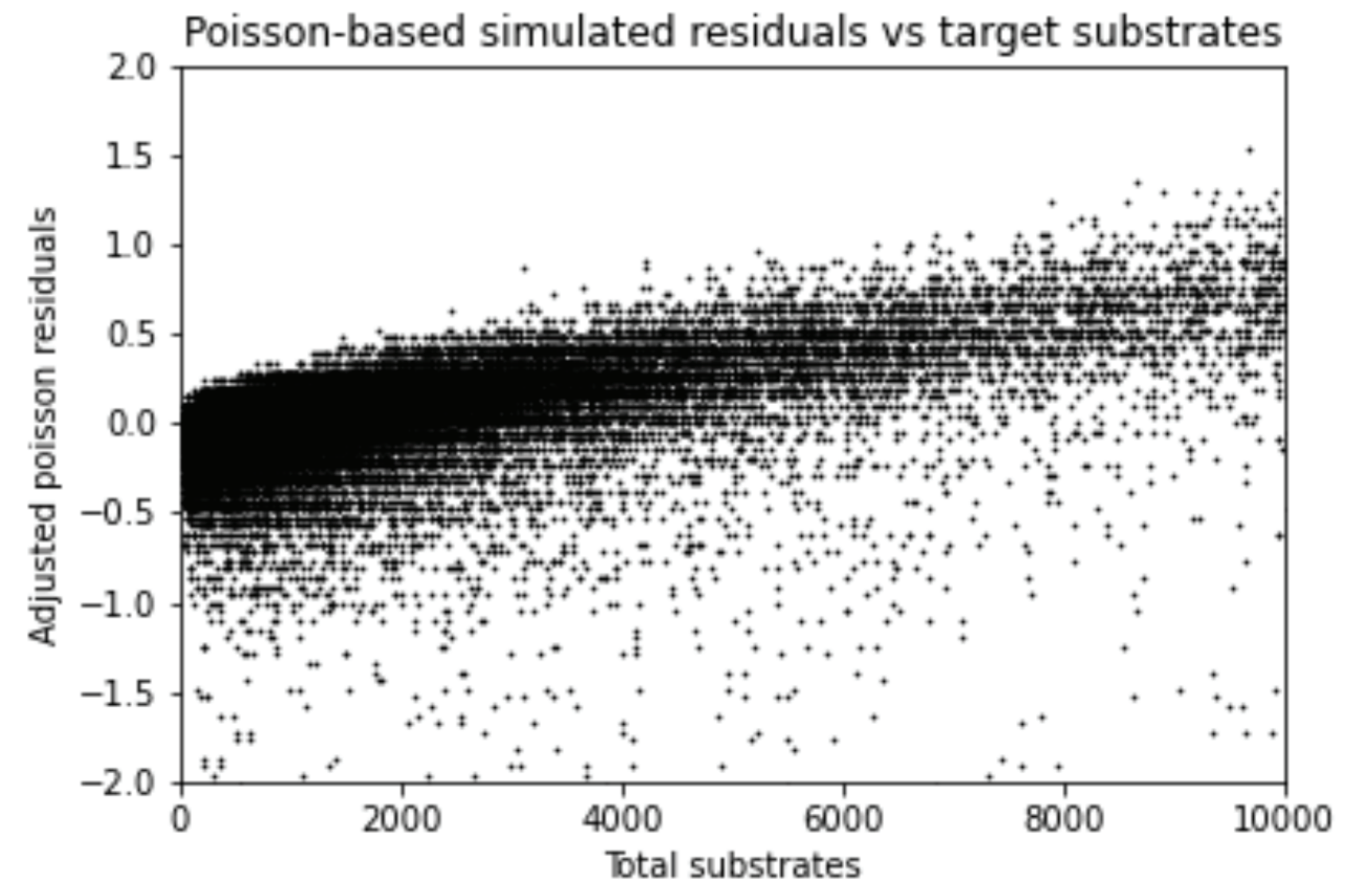

Supplement: Supplementary file 1 — Additional file 1: Figure S1. Validation of the zero-truncated Poisson model. a Simulated edit count distribution matches actual edit count distribution well. b Residuals at lower coverage windows exhibit little correlation to coverage. It is worth noting that although the model loses fidelity in regions at extremely highly covered windows (c), the resulting overestimation of expected edit counts in such areas will tend to lead to under-calling of regions rather than false positives, erring on the conservative side to increase precision at the expense of recall [file 12859_2023_5452_MOESM1_ESM.pdf]

A

Fraction of FLARE clusters overlapping fusion eCLIP:  
Enrichment compared to WT

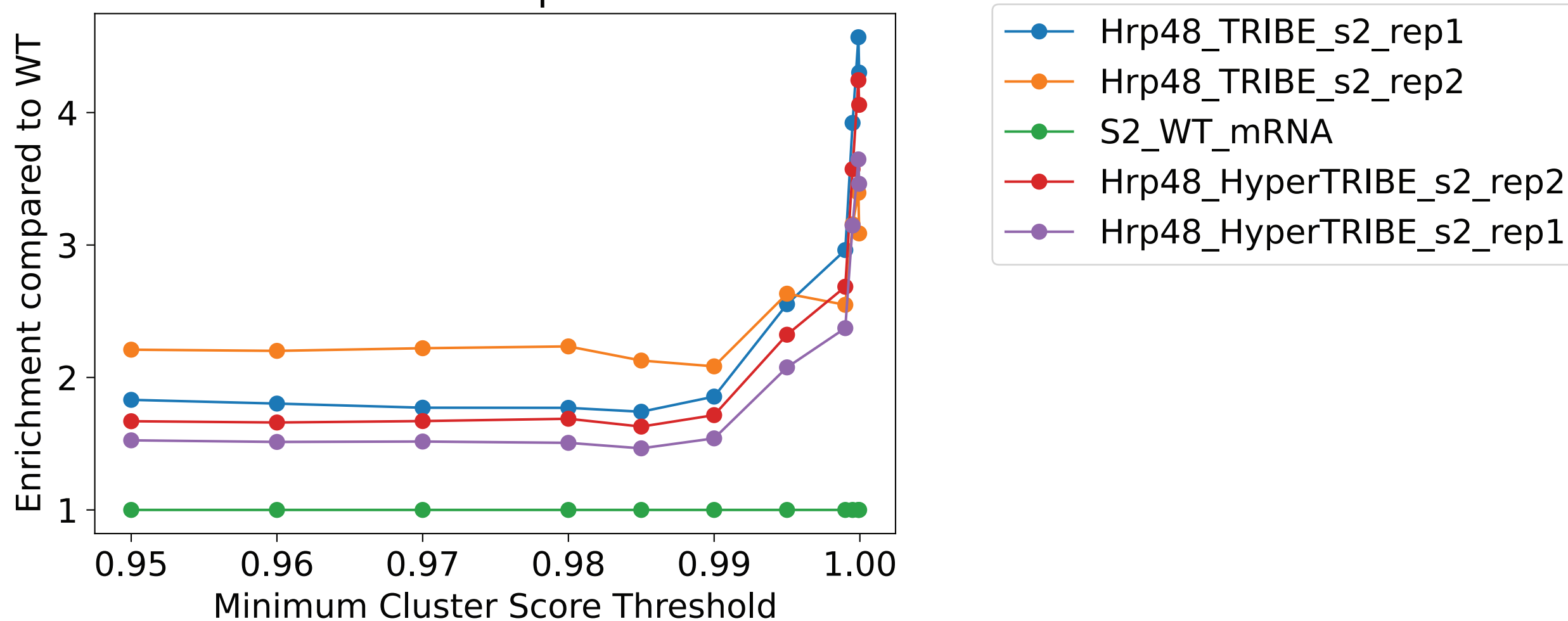

Supplement: Supplementary file 3 — Additional file 3: Figure S3. TRIBE and HyperTRIBE datasets are amenable to FLARE analysis. a There is an enrichment for overlap with Hrp48 CLIP peaks of Hrp48-TRIBE and Hrp48-HyperTRIBE, when normalized to WT overlap [file 12859_2023_5452_MOESM3_ESM.pdf]
