# Supplementary material for: FLARE: a fast and flexible workflow for identifying RNA editing foci
Source: BMC Bioinformatics. 2023 Oct 2;24:370. doi: 10.1186/s12859-023-05452-4 (PMC10544219; doi:10.1186/s12859-023-05452-4)
Supplement: Supplementary file 2 — Additional file 2: Figure S2. FLARE cluster properties and comparison to site-based approach. a The correlation of window edit fraction with window precision, which is expected to be both positive and uncorrelated to window read depth, only stabilizes above a read depth of approximately 50 reads. b Generating 50 base pair windows surrounding individual SAILOR sites, filtered by individual SAILOR site score exclusively, results in lower specificity than the approach employed by FLARE. c A site-based approach also yields lower sensitivity than the cluster-based approach. d Expanding RBOX2 FLARE clusters leads to higher rates of eCLIP or canonical RBFOX2 motif overlap. e FLARE clusters overlapping eCLIP are slightly likelier than ones not overlapping eCLIP to contain secondary RBFOX2 motifs. f A higher fraction of eCLIP overlapping clusters contain secondary motifs than control motifs. g A higher fraction of non-eCLIP overlapping clusters contain secondary motifs than control motifs. h Between approximately 25% and 40% of edit sites can be found within peaks depending on score threshold. i The cluster length distribution with 30 bp windows has a long tail but is centered around approximately 100 bp [file 12859_2023_5452_MOESM2_ESM.pdf]

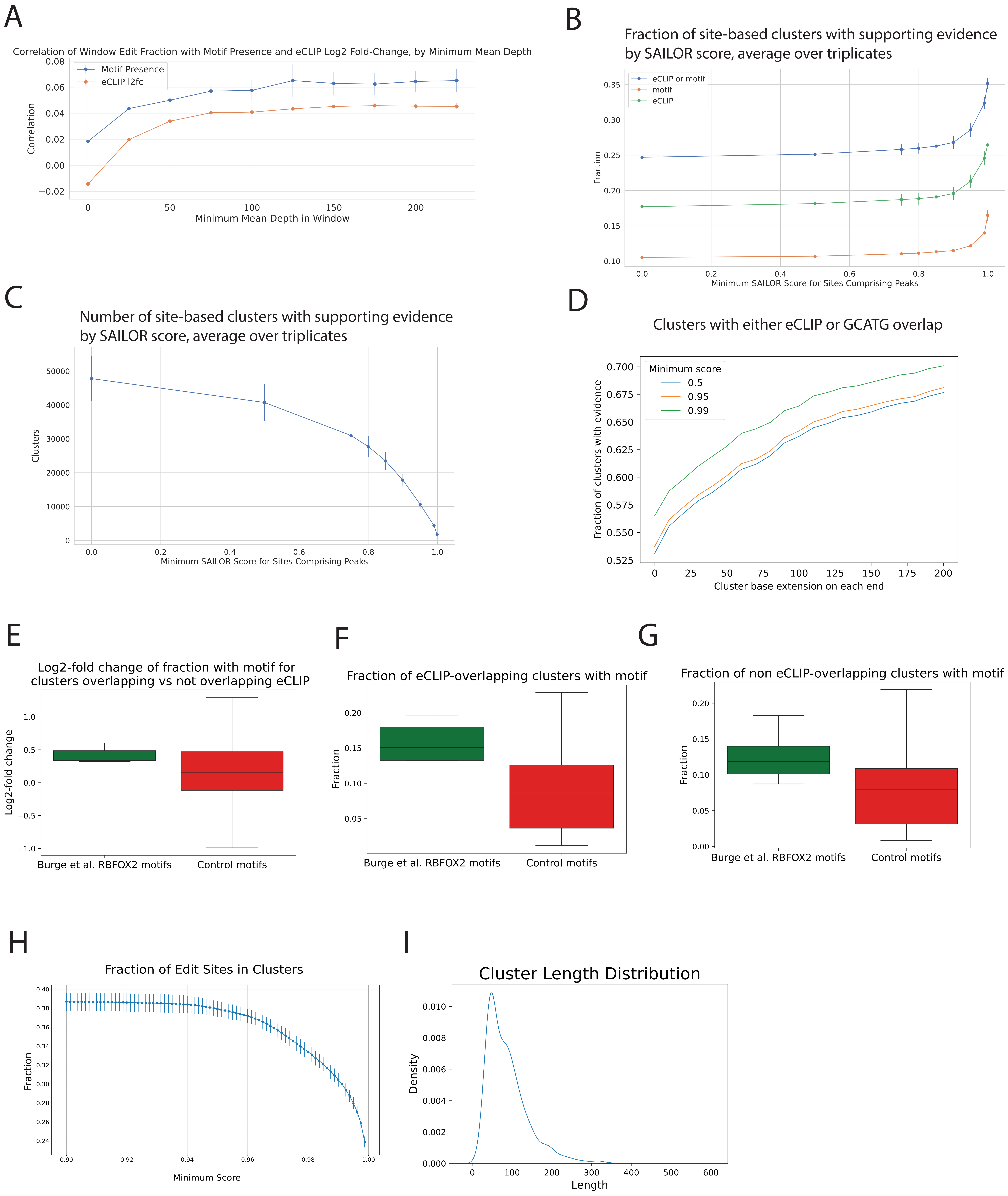
A
Correlation of Window Edit Fraction with Motif Presence and eCLIP Log2 Fold-Change, by Minimum Mean Depth
Motif Presence
eCLIP l2fc
Correlation
Minimum Mean Depth in Window
B
Fraction of site-based clusters with supporting evidence by SAILOR score, average over triplicates
eCLIP or motif
motif
eCLIP
Fraction
Minimum SAILOR Score for Sites Comprising Peaks
C
Number of site-based clusters with supporting evidence by SAILOR score, average over triplicates
Clusters
Minimum SAILOR Score for Sites Comprising Peaks
D
Clusters with either eCLIP or GCATG overlap
Minimum score
0.5
0.95
0.99
Fraction of clusters with evidence
Cluster base extension on each end
E
Log2-fold change of fraction with motif for clusters overlapping vs not overlapping eCLIP
Log2-fold change
Burge et al. RBFOX2 motifs
Control motifs
F
Fraction of eCLIP-overlapping clusters with motif
Fraction
Burge et al. RBFOX2 motifs
Control motifs
G
Fraction of non eCLIP-overlapping clusters with motif
Fraction
Burge et al. RBFOX2 motifs
Control motifs
H
Fraction of Edit Sites in Clusters
Fraction
Minimum Score
I
Cluster Length Distribution
Density
Length
