# Supplementary material for: FLARE: a fast and flexible workflow for identifying RNA editing foci
Source: BMC Bioinformatics. 2023 Oct 2;24:370. doi: 10.1186/s12859-023-05452-4 (PMC10544219; doi:10.1186/s12859-023-05452-4)

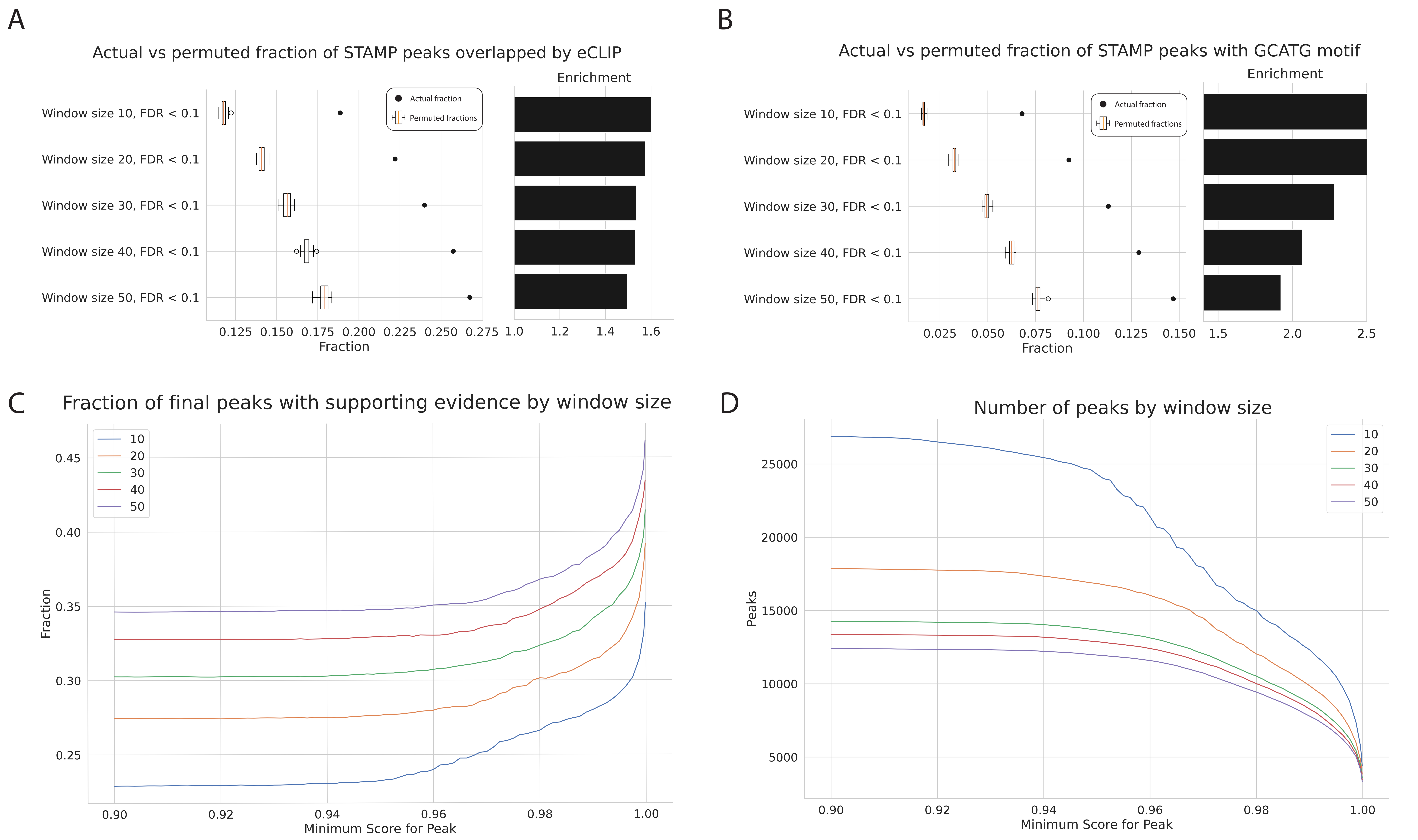

E

$$\text{Efficiency score}\,(\textit{window size}) = \frac{\text{sites per cluster}_{\textit{window size}}}{\text{total area covered by clusters}_{\textit{window size}}}$$

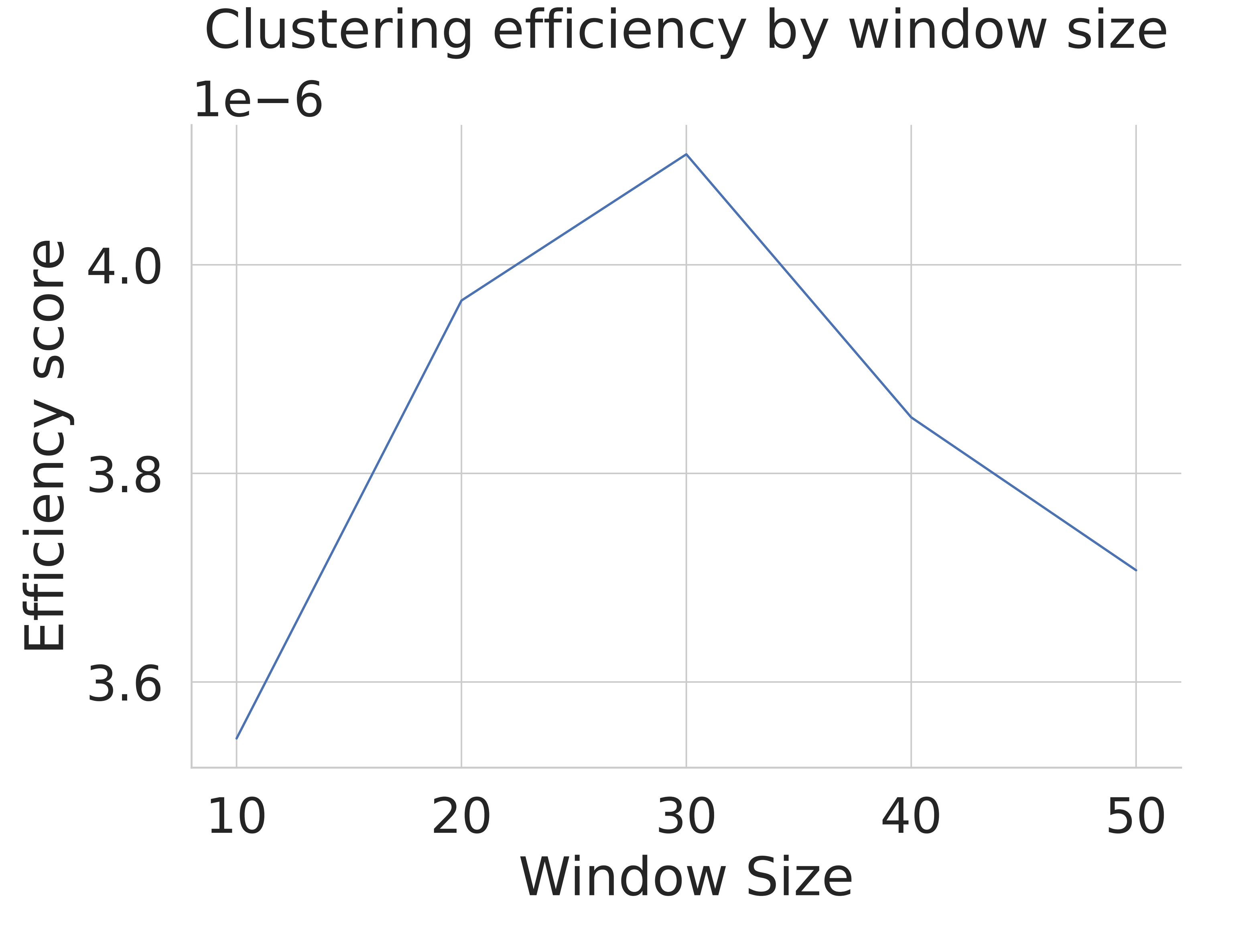

Supplement: Supplementary file 4 — Additional file 4: Figure S4. Window size affects sensitivity and statistical significance—graphs produced using a single replicate. a Larger windows yield more precise but slightly less statistically significant RBFOX2 clusters based on eCLIP overlap. b Larger windows yield more precise but far less statistically significant RBFOX2 clusters based on eCLIP overlap. c Marginal gains in precision from larger window sizes decrease for each size increase. d As window size increases, neighboring small clusters merge to form larger clusters, but this merging and the resultant decrease in total clusters is less dramatic at higher values. e The “efficiency score” metric can be serve as a guide to tune window sizes for optimal clustering [file 12859_2023_5452_MOESM4_ESM.pdf]
